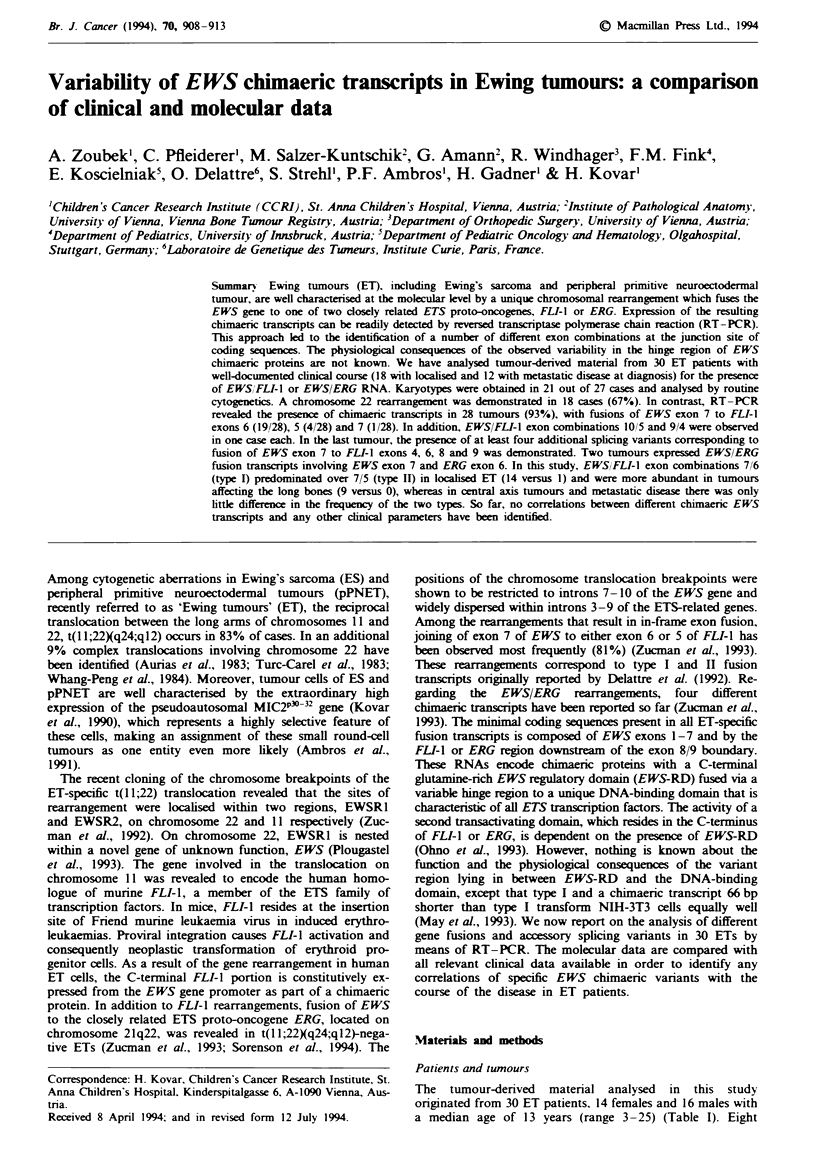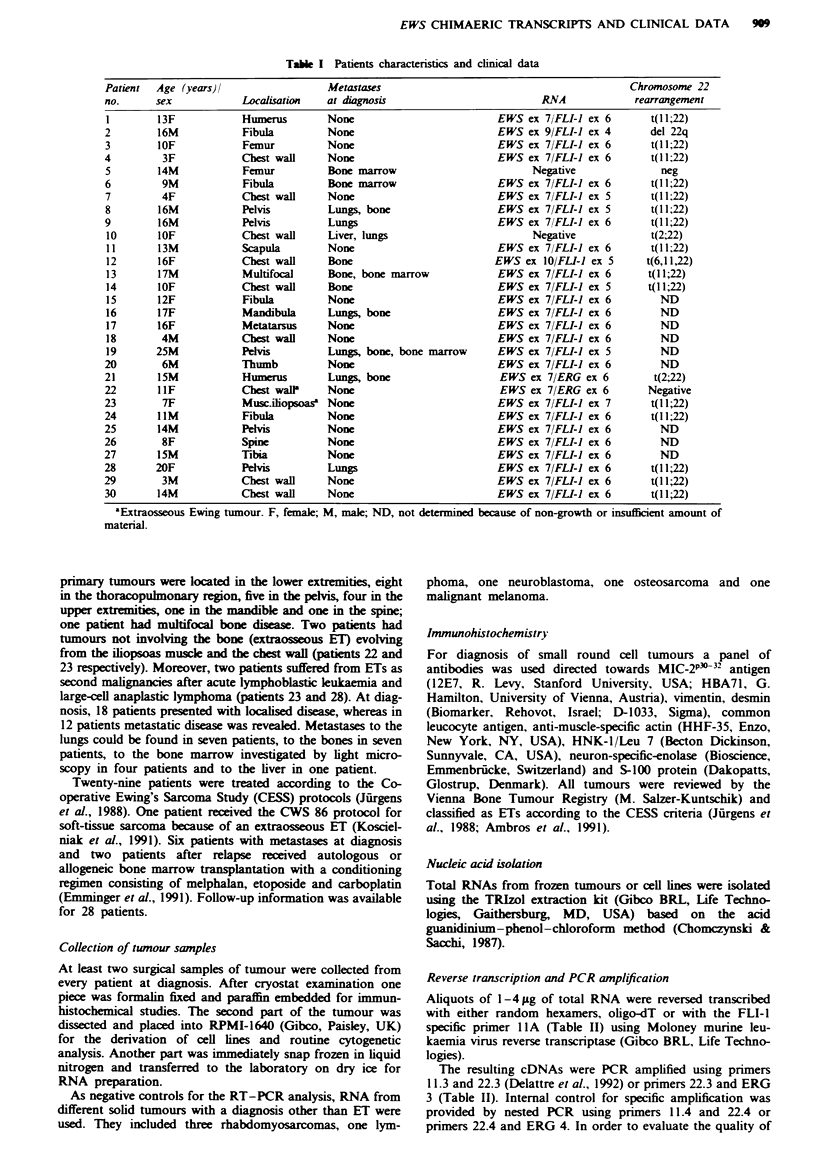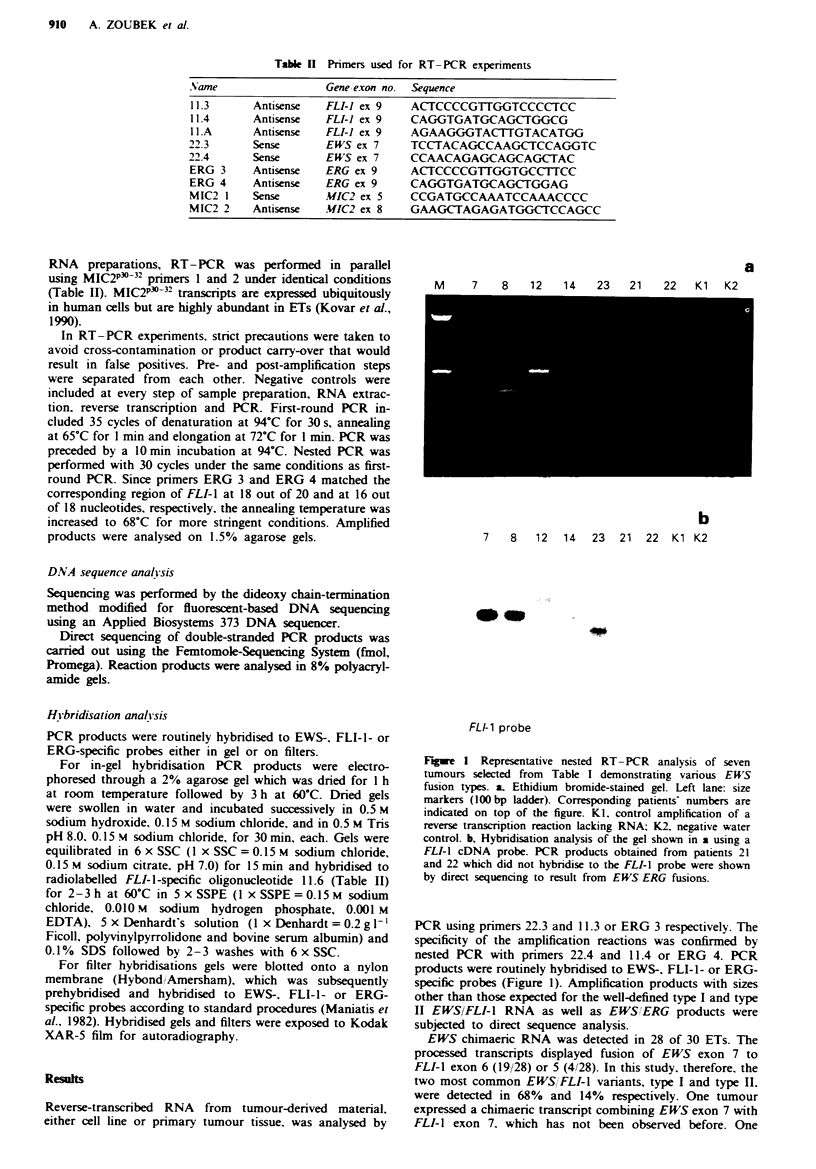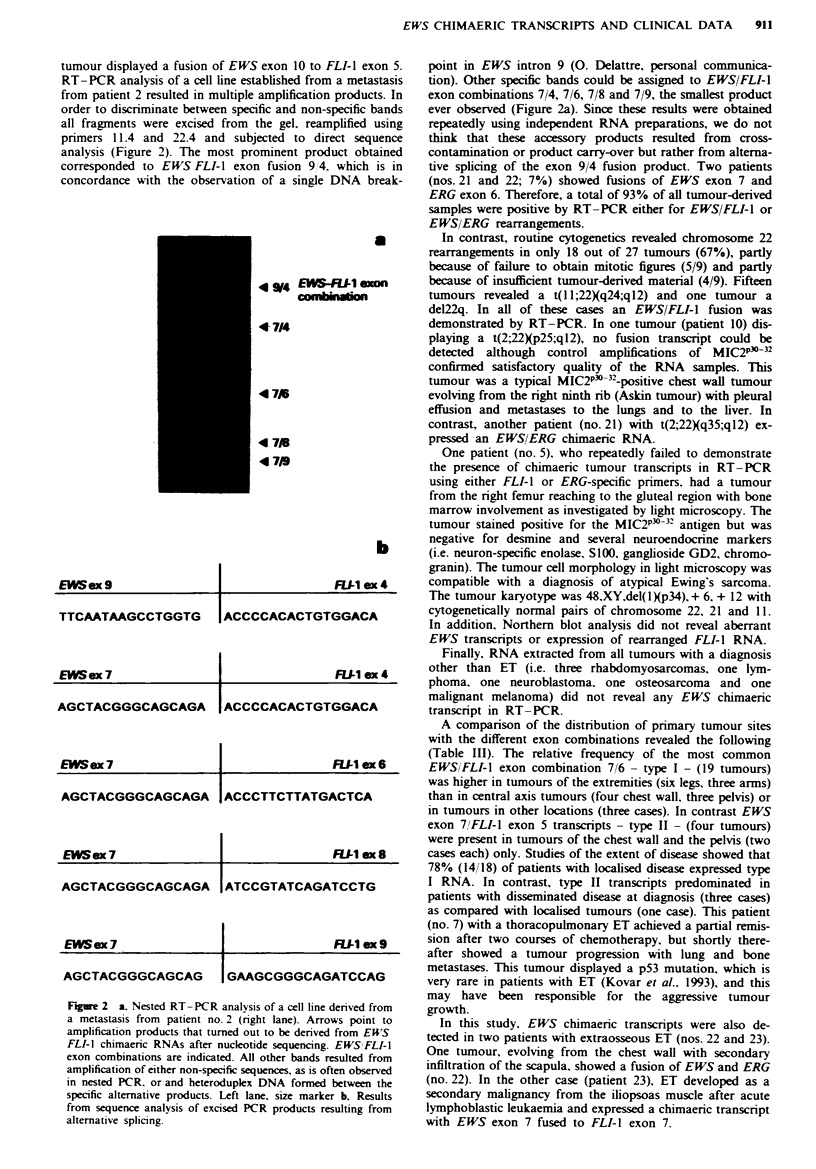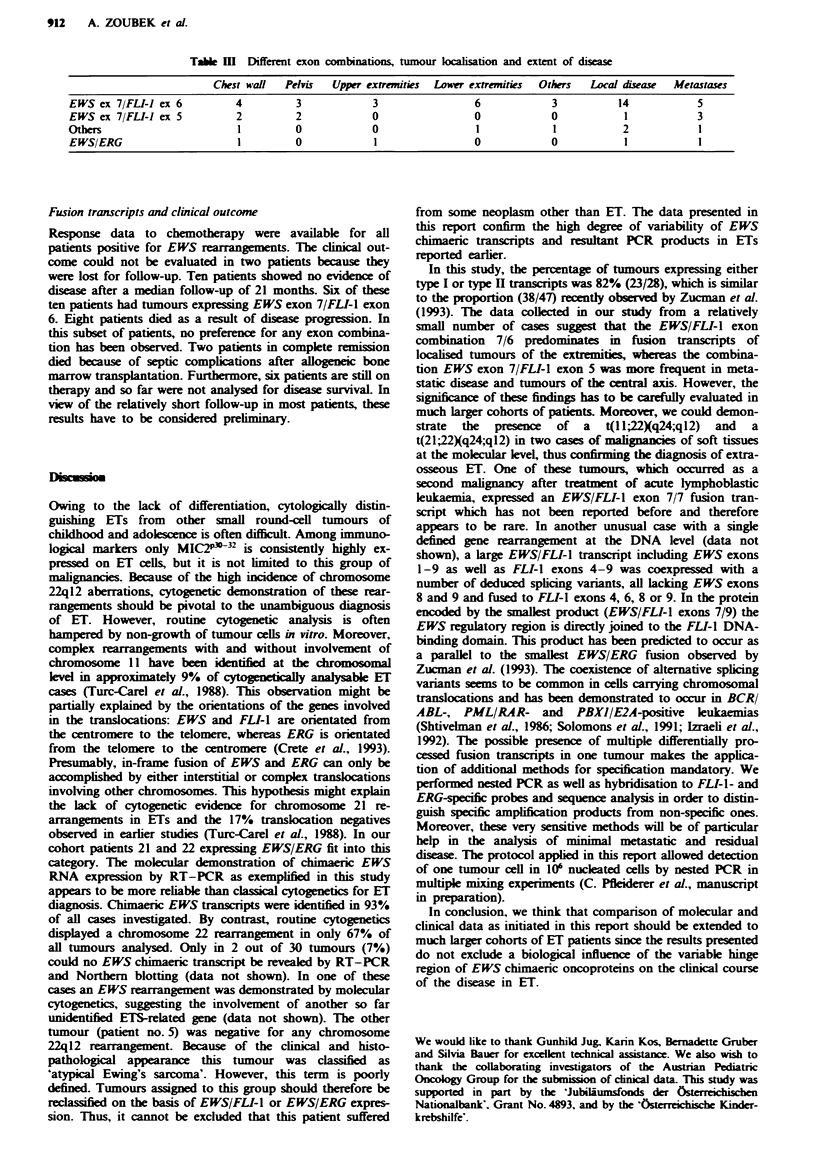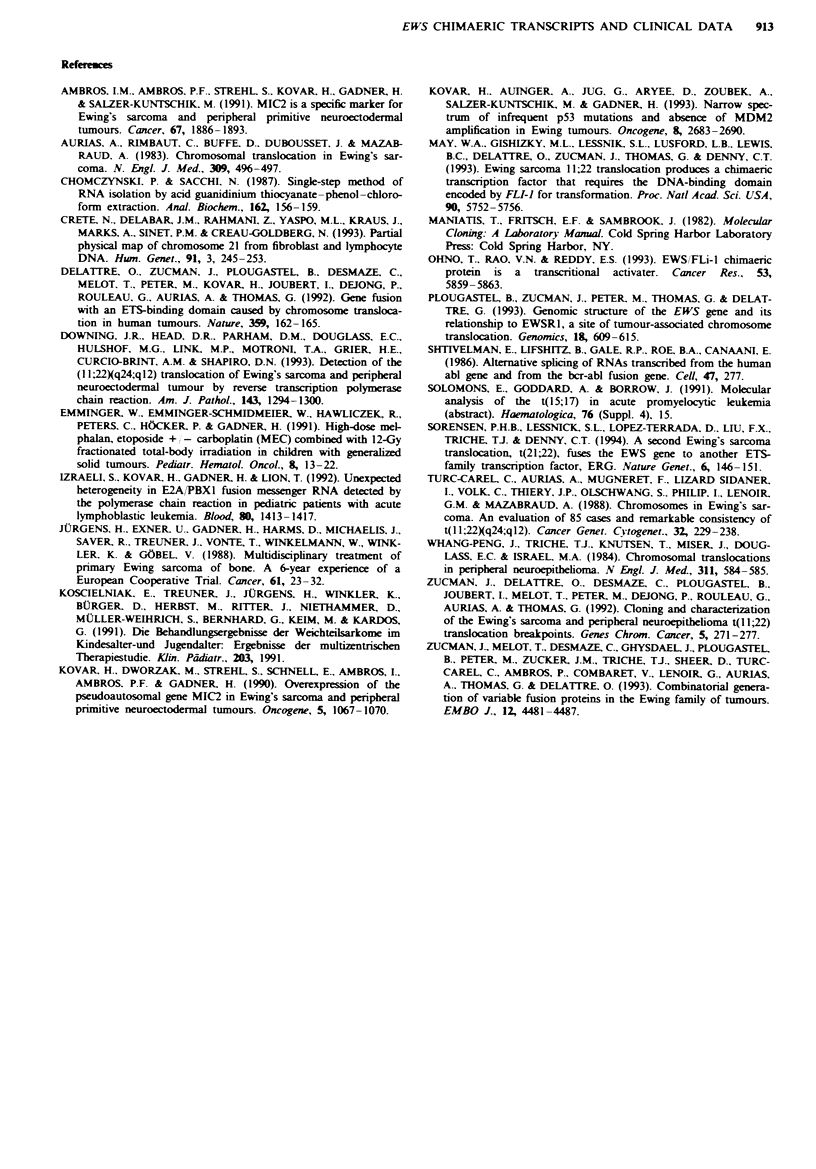# Variability of EWS chimaeric transcripts in Ewing tumours: a comparison of clinical and molecular data.

**DOI:** 10.1038/bjc.1994.419

**Published:** 1994-11

**Authors:** A. Zoubek, C. Pfleiderer, M. Salzer-Kuntschik, G. Amann, R. Windhager, F. M. Fink, E. Koscielniak, O. Delattre, S. Strehl, P. F. Ambros

**Affiliations:** Children's Cancer Research Institute (CCRI), St. Anna Children's Hospital, Vienna, Austria.

## Abstract

**Images:**


Br. J. Cancer (1994), 70, 908-913 © Macmillan Press Ltd., 1994

### Variability of *EWS* chimaeric transcripts in Ewing tumours: a comparison of clinical and molecular data

A. Zoubek[1], C. Pfleiderer[1], M. Salzer-Kuntschik[2], G. Amann[2], R. Windhager[3], F.M. Fink[4], E. Koscielniak[5], O. Delattre[6], S. Strehl[1], P.F. Ambros[1], H. Gadner[1] & H. Kovar[1]

[1]*Children's Cancer Research Institute (CCRI), St. Anna Children's Hospital, Vienna, Austria;* [2]*Institute of Pathological Anatomy, University of Vienna, Vienna Bone Tumour Registry, Austria;* [3]*Department of Orthopedic Surgery, University of Vienna, Austria;* [4]*Department of Pediatrics, University of Innsbruck, Austria;* [5]*Department of Pediatric Oncology and Hematology, Olgahospital, Stuttgart, Germany;* [6]*Laboratoire de Genetique des Tumeurs, Institute Curie, Paris, France.*

**Summary** Ewing tumours (ET), including Ewing's sarcoma and peripheral primitive neuroectodermal tumour, are well characterised at the molecular level by a unique chromosomal rearrangement which fuses the *EWS* gene to one of two closely related *ETS* proto-oncogenes, *FLI-1* or *ERG*. Expression of the resulting chimaeric transcripts can be readily detected by reversed transcriptase polymerase chain reaction (RT-PCR). This approach led to the identification of a number of different exon combinations at the junction site of coding sequences. The physiological consequences of the observed variability in the hinge region of *EWS* chimaeric proteins are not known. We have analysed tumour-derived material from 30 ET patients with well-documented clinical course (18 with localised and 12 with metastatic disease at diagnosis) for the presence of *EWS/FLI-1* or *EWS/ERG* RNA. Karyotypes were obtained in 21 out of 27 cases and analysed by routine cytogenetics. A chromosome 22 rearrangement was demonstrated in 18 cases (67%). In contrast, RT-PCR revealed the presence of chimaeric transcripts in 28 tumours (93%), with fusions of *EWS* exon 7 to *FLI-1* exons 6 (19/28), 5 (4/28) and 7 (1/28). In addition, *EWS/FLI-1* exon combinations 10/5 and 9/4 were observed in one case each. In the last tumour, the presence of at least four additional splicing variants corresponding to fusion of *EWS* exon 7 to *FLI-1* exons 4, 6, 8 and 9 was demonstrated. Two tumours expressed *EWS/ERG* fusion transcripts involving *EWS* exon 7 and *ERG* exon 6. In this study, *EWS/FLI-1* exon combinations 7/6 (type I) predominated over 7/5 (type II) in localised ET (14 versus 1) and were more abundant in tumours affecting the long bones (9 versus 0), whereas in central axis tumours and metastatic disease there was only little difference in the frequency of the two types. So far, no correlations between different chimaeric *EWS* transcripts and any other clinical parameters have been identified.

Among cytogenetic aberrations in Ewing's sarcoma (ES) and peripheral primitive neuroectodermal tumours (pPNET), recently referred to as 'Ewing tumours' (ET), the reciprocal translocation between the long arms of chromosomes 11 and 22, t(11;22)(q24;q12) occurs in 83% of cases. In an additional 9% complex translocations involving chromosome 22 have been identified (Aurias *et al.*, 1983; Turc-Carel *et al.*, 1983; Whang-Peng *et al.*, 1984). Moreover, tumour cells of ES and pPNET are well characterised by the extraordinary high expression of the pseudoautosomal MIC2$^{p30-32}$ gene (Kovar *et al.*, 1990), which represents a highly selective feature of these cells, making an assignment of these small round-cell tumours as one entity even more likely (Ambros *et al.*, 1991).

The recent cloning of the chromosome breakpoints of the ET-specific t(11;22) translocation revealed that the sites of rearrangement were localised within two regions, EWSR1 and EWSR2, on chromosome 22 and 11 respectively (Zucman *et al.*, 1992). On chromosome 22, EWSR1 is nested within a novel gene of unknown function, *EWS* (Plougastel *et al.*, 1993). The gene involved in the translocation on chromosome 11 was revealed to encode the human homologue of murine *FLI-1*, a member of the ETS family of transcription factors. In mice, *FLI-1* resides at the insertion site of Friend murine leukaemia virus in induced erythroleukaemias. Proviral integration causes *FLI-1* activation and consequently neoplastic transformation of erythroid progenitor cells. As a result of the gene rearrangement in human ET cells, the C-terminal *FLI-1* portion is constitutively expressed from the *EWS* gene promoter as part of a chimaeric protein. In addition to *FLI-1* rearrangements, fusion of *EWS* to the closely related ETS proto-oncogene *ERG*, located on chromosome 21q22, was revealed in t(11;22)(q24;q12)-negative ETs (Zucman *et al.*, 1993; Sorenson *et al.*, 1994). The positions of the chromosome translocation breakpoints were shown to be restricted to introns 7-10 of the *EWS* gene and widely dispersed within introns 3-9 of the ETS-related genes. Among the rearrangements that result in in-frame exon fusion, joining of exon 7 of *EWS* to either exon 6 or 5 of *FLI-1* has been observed most frequently (81%) (Zucman *et al.*, 1993). These rearrangements correspond to type I and II fusion transcripts originally reported by Delattre *et al.* (1992). Regarding the *EWS/ERG* rearrangements, four different chimaeric transcripts have been reported so far (Zucman *et al.*, 1993). The minimal coding sequences present in all ET-specific fusion transcripts is composed of *EWS* exons 1-7 and by the *FLI-1* or *ERG* region downstream of the exon 8/9 boundary. These RNAs encode chimaeric proteins with a C-terminal glutamine-rich *EWS* regulatory domain (*EWS*-RD) fused via a variable hinge region to a unique DNA-binding domain that is characteristic of all *ETS* transcription factors. The activity of a second transactivating domain, which resides in the C-terminus of *FLI-1* or *ERG*, is dependent on the presence of *EWS*-RD (Ohno *et al.*, 1993). However, nothing is known about the function and the physiological consequences of the variant region lying in between *EWS*-RD and the DNA-binding domain, except that type I and a chimaeric transcript 66 bp shorter than type I transform NIH-3T3 cells equally well (May *et al.*, 1993). We now report on the analysis of different gene fusions and accessory splicing variants in 30 ETs by means of RT-PCR. The molecular data are compared with all relevant clinical data available in order to identify any correlations of specific *EWS* chimaeric variants with the course of the disease in ET patients.

### Materials and methods

### *Patients and tumours*

The tumour-derived material analysed in this study originated from 30 ET patients, 14 females and 16 males with a median age of 13 years (range 3-25) (Table I). Eight

Correspondence: H. Kovar, Children's Cancer Research Institute, St. Anna Children's Hospital, Kinderspitalgasse 6, A-1090 Vienna, Austria.

Received 8 April 1994; and in revised form 12 July 1994.

**Table I** Patients characteristics and clinical data

| *Patient no.* | *Age (years)/ sex* | *Localisation* | *Metastases at diagnosis* | *RNA* | *Chromosome 22 rearrangement* |
|---|---|---|---|---|---|
| 1 | 13F | Humerus | None | *EWS* ex 7/*FLI-1* ex 6 | t(11;22) |
| 2 | 16M | Fibula | None | *EWS* ex 9/*FLI-1* ex 4 | del 22q |
| 3 | 10F | Femur | None | *EWS* ex 7/*FLI-1* ex 6 | t(11;22) |
| 4 | 3F | Chest wall | None | *EWS* ex 7/*FLI-1* ex 6 | t(11;22) |
| 5 | 14M | Femur | Bone marrow | Negative | neg |
| 6 | 9M | Fibula | Bone marrow | *EWS* ex 7/*FLI-1* ex 6 | t(11;22) |
| 7 | 4F | Chest wall | None | *EWS* ex 7/*FLI-1* ex 5 | t(11;22) |
| 8 | 16M | Pelvis | Lungs, bone | *EWS* ex 7/*FLI-1* ex 5 | t(11;22) |
| 9 | 16M | Pelvis | Lungs | *EWS* ex 7/*FLI-1* ex 6 | t(11;22) |
| 10 | 10F | Chest wall | Liver, lungs | Negative | t(2;22) |
| 11 | 13M | Scapula | None | *EWS* ex 7/*FLI-1* ex 6 | t(11;22) |
| 12 | 16F | Chest wall | Bone | *EWS* ex 10/*FLI-1* ex 5 | t(6,11,22) |
| 13 | 17M | Multifocal | Bone, bone marrow | *EWS* ex 7/*FLI-1* ex 6 | t(11;22) |
| 14 | 10F | Chest wall | Bone | *EWS* ex 7/*FLI-1* ex 5 | t(11;22) |
| 15 | 12F | Fibula | None | *EWS* ex 7/*FLI-1* ex 6 | ND |
| 16 | 17F | Mandibula | Lungs, bone | *EWS* ex 7/*FLI-1* ex 6 | ND |
| 17 | 16F | Metatarsus | None | *EWS* ex 7/*FLI-1* ex 6 | ND |
| 18 | 4M | Chest wall | None | *EWS* ex 7/*FLI-1* ex 6 | ND |
| 19 | 25M | Pelvis | Lungs, bone, bone marrow | *EWS* ex 7/*FLI-1* ex 5 | ND |
| 20 | 6M | Thumb | None | *EWS* ex 7/*FLI-1* ex 6 | ND |
| 21 | 15M | Humerus | Lungs, bone | *EWS* ex 7/*ERG* ex 6 | t(2;22) |
| 22 | 11F | Chest wall[a] | None | *EWS* ex 7/*ERG* ex 6 | Negative |
| 23 | 7F | Musc.iliopsoas[a] | None | *EWS* ex 7/*FLI-1* ex 7 | t(11;22) |
| 24 | 11M | Fibula | None | *EWS* ex 7/*FLI-1* ex 6 | t(11;22) |
| 25 | 14M | Pelvis | None | *EWS* ex 7/*FLI-1* ex 6 | ND |
| 26 | 8F | Spine | None | *EWS* ex 7/*FLI-1* ex 6 | ND |
| 27 | 15M | Tibia | None | *EWS* ex 7/*FLI-1* ex 6 | ND |
| 28 | 20F | Pelvis | Lungs | *EWS* ex 7/*FLI-1* ex 6 | t(11;22) |
| 29 | 3M | Chest wall | None | *EWS* ex 7/*FLI-1* ex 6 | t(11;22) |
| 30 | 14M | Chest wall | None | *EWS* ex 7/*FLI-1* ex 6 | t(11;22) |

[a]Extraosseous Ewing tumour. F, female; M, male; ND, not determined because of non-growth or insufficient amount of material.

primary tumours were located in the lower extremities, eight in the thoracopulmonary region, five in the pelvis, four in the upper extremities, one in the mandible and one in the spine; one patient had multifocal bone disease. Two patients had tumours not involving the bone (extraosseous ET) evolving from the iliopsoas muscle and the chest wall (patients 22 and 23 respectively). Moreover, two patients suffered from ETs as second malignancies after acute lymphoblastic leukaemia and large-cell anaplastic lymphoma (patients 23 and 28). At diagnosis, 18 patients presented with localised disease, whereas in 12 patients metastatic disease was revealed. Metastases to the lungs could be found in seven patients, to the bones in seven patients, to the bone marrow investigated by light microscopy in four patients and to the liver in one patient.

Twenty-nine patients were treated according to the Co-operative Ewing's Sarcoma Study (CESS) protocols (Jürgens *et al.*, 1988). One patient received the CWS 86 protocol for soft-tissue sarcoma because of an extraosseous ET (Koscielniak *et al.*, 1991). Six patients with metastases at diagnosis and two patients after relapse received autologous or allogeneic bone marrow transplantation with a conditioning regimen consisting of melphalan, etoposide and carboplatin (Emminger *et al.*, 1991). Follow-up information was available for 28 patients.

### *Collection of tumour samples*

At least two surgical samples of tumour were collected from every patient at diagnosis. After cryostat examination one piece was formalin fixed and paraffin embedded for immunhistochemical studies. The second part of the tumour was dissected and placed into RPMI-1640 (Gibco, Paisley, UK) for the derivation of cell lines and routine cytogenetic analysis. Another part was immediately snap frozen in liquid nitrogen and transferred to the laboratory on dry ice for RNA preparation.

As negative controls for the RT–PCR analysis, RNA from different solid tumours with a diagnosis other than ET were used. They included three rhabdomyosarcomas, one lymphoma, one neuroblastoma, one osteosarcoma and one malignant melanoma.

### *Immunohistochemistry*

For diagnosis of small round cell tumours a panel of antibodies was used directed towards MIC-2$^{p30-32}$ antigen (12E7, R. Levy, Stanford University, USA; HBA71, G. Hamilton, University of Vienna, Austria), vimentin, desmin (Biomarker, Rehovot, Israel; D-1033, Sigma), common leucocyte antigen, anti-muscle-specific actin (HHF-35, Enzo, New York, NY, USA), HNK-1/Leu 7 (Becton Dickinson, Sunnyvale, CA, USA), neuron-specific-enolase (Bioscience, Emmenbrücke, Switzerland) and S-100 protein (Dakopatts, Glostrup, Denmark). All tumours were reviewed by the Vienna Bone Tumour Registry (M. Salzer-Kuntschik) and classified as ETs according to the CESS criteria (Jürgens *et al.*, 1988; Ambros *et al.*, 1991).

### *Nucleic acid isolation*

Total RNAs from frozen tumours or cell lines were isolated using the TRIzol extraction kit (Gibco BRL, Life Technologies, Gaithersburg, MD, USA) based on the acid guanidinium–phenol–chloroform method (Chomczynski & Sacchi, 1987).

### *Reverse transcription and PCR amplification*

Aliquots of 1–4 μg of total RNA were reversed transcribed with either random hexamers, oligo-dT or with the FLI-1 specific primer 11A (Table II) using Moloney murine leukaemia virus reverse transcriptase (Gibco BRL, Life Technologies).

The resulting cDNAs were PCR amplified using primers 11.3 and 22.3 (Delattre *et al.*, 1992) or primers 22.3 and ERG 3 (Table II). Internal control for specific amplification was provided by nested PCR using primers 11.4 and 22.4 or primers 22.4 and ERG 4. In order to evaluate the quality of

**Table II** Primers used for RT-PCR experiments

| *Name* | | *Gene/exon no.* | *Sequence* |
|---|---|---|---|
| 11.3 | Antisense | *FLI-1* ex 9 | ACTCCCCGTTGGTCCCCTCC |
| 11.4 | Antisense | *FLI-1* ex 9 | CAGGTGATGCAGCTGGCG |
| 11.A | Antisense | *FLI-1* ex 9 | AGAAGGGTACTTGTACATGG |
| 22.3 | Sense | *EWS* ex 7 | TCCTACAGCCAAGCTCCAGGTC |
| 22.4 | Sense | *EWS* ex 7 | CCAACAGAGCAGCAGCTAC |
| ERG 3 | Antisense | *ERG* ex 9 | ACTCCCCGTTGGTGCCTTCC |
| ERG 4 | Antisense | *ERG* ex 9 | CAGGTGATGCAGCTGGAG |
| MIC2 1 | Sense | *MIC2* ex 5 | CCGATGCCAAATCCAAACCCC |
| MIC2 2 | Antisense | *MIC2* ex 8 | GAAGCTAGAGATGGCTCCAGCC |

RNA preparations, RT-PCR was performed in parallel using MIC2$^{p30-32}$ primers 1 and 2 under identical conditions (Table II). MIC2$^{p30-32}$ transcripts are expressed ubiquitously in human cells but are highly abundant in ETs (Kovar *et al.*, 1990).

In RT-PCR experiments, strict precautions were taken to avoid cross-contamination or product carry-over that would result in false positives. Pre- and post-amplification steps were separated from each other. Negative controls were included at every step of sample preparation, RNA extraction, reverse transcription and PCR. First-round PCR included 35 cycles of denaturation at 94°C for 30 s, annealing at 65°C for 1 min and elongation at 72°C for 1 min. PCR was preceded by a 10 min incubation at 94°C. Nested PCR was performed with 30 cycles under the same conditions as first-round PCR. Since primers ERG 3 and ERG 4 matched the corresponding region of *FLI*-1 at 18 out of 20 and at 16 out of 18 nucleotides, respectively, the annealing temperature was increased to 68°C for more stringent conditions. Amplified products were analysed on 1.5% agarose gels.

### *DNA sequence analysis*

Sequencing was performed by the dideoxy chain-termination method modified for fluorescent-based DNA sequencing using an Applied Biosystems 373 DNA sequencer.

Direct sequencing of double-stranded PCR products was carried out using the Femtomole-Sequencing System (fmol, Promega). Reaction products were analysed in 8% polyacrylamide gels.

### *Hybridisation analysis*

PCR products were routinely hybridised to EWS-, FLI-1- or ERG-specific probes either in gel or on filters.

For in-gel hybridisation PCR products were electrophoresed through a 2% agarose gel which was dried for 1 h at room temperature followed by 3 h at 60°C. Dried gels were swollen in water and incubated successively in 0.5 M sodium hydroxide, 0.15 M sodium chloride, and in 0.5 M Tris pH 8.0, 0.15 M sodium chloride, for 30 min, each. Gels were equilibrated in 6 × SSC (1 × SSC = 0.15 M sodium chloride, 0.15 M sodium citrate, pH 7.0) for 15 min and hybridised to radiolabelled *FLI*-1-specific oligonucleotide 11.6 (Table II) for 2-3 h at 60°C in 5 × SSPE (1 × SSPE = 0.15 M sodium chloride, 0.010 M sodium hydrogen phosphate, 0.001 M EDTA), 5 × Denhardt's solution (1 × Denhardt = 0.2 g $l^{-1}$ Ficoll, polyvinylpyrrolidone and bovine serum albumin) and 0.1% SDS followed by 2-3 washes with 6 × SSC.

For filter hybridisations gels were blotted onto a nylon membrane (Hybond/Amersham), which was subsequently prehybridised and hybridised to EWS-, FLI-1- or ERG-specific probes according to standard procedures (Maniatis *et al.*, 1982). Hybridised gels and filters were exposed to Kodak XAR-5 film for autoradiography.

### Results

Reverse-transcribed RNA from tumour-derived material, either cell line or primary tumour tissue, was analysed by

**Figure 1** Representative nested RT-PCR analysis of seven tumours selected from Table I demonstrating various *EWS* fusion types. **a**, Ethidium bromide-stained gel. Left lane: size markers (100 bp ladder). Corresponding patients' numbers are indicated on top of the figure. K1, control amplification of a reverse transcription reaction lacking RNA; K2, negative water control. **b**, Hybridisation analysis of the gel shown in **a** using a *FLI*-1 cDNA probe. PCR products obtained from patients 21 and 22 which did not hybridise to the *FLI*-1 probe were shown by direct sequencing to result from *EWS/ERG* fusions.

PCR using primers 22.3 and 11.3 or ERG 3 respectively. The specificity of the amplification reactions was confirmed by nested PCR with primers 22.4 and 11.4 or ERG 4. PCR products were routinely hybridised to EWS-, FLI-1- or ERG-specific probes (Figure 1). Amplification products with sizes other than those expected for the well-defined type I and type II *EWS/FLI*-1 RNA as well as *EWS/ERG* products were subjected to direct sequence analysis.

*EWS* chimaeric RNA was detected in 28 of 30 ETs. The processed transcripts displayed fusion of *EWS* exon 7 to *FLI*-1 exon 6 (19/28) or 5 (4/28). In this study, therefore, the two most common *EWS/FLI*-1 variants, type I and type II, were detected in 68% and 14% respectively. One tumour expressed a chimaeric transcript combining *EWS* exon 7 with *FLI*-1 exon 7, which has not been observed before. One tumour displayed a fusion of *EWS* exon 10 to *FLI*-1 exon 5. RT-PCR analysis of a cell line established from a metastasis from patient 2 resulted in multiple amplification products. In order to discriminate between specific and non-specific bands all fragments were excised from the gel, reamplified using primers 11.4 and 22.4 and subjected to direct sequence analysis (Figure 2). The most prominent product obtained corresponded to *EWS/FLI*-1 exon fusion 9/4, which is in concordance with the observation of a single DNA breakpoint in *EWS* intron 9 (O. Delattre, personal communication). Other specific bands could be assigned to *EWS/FLI*-1 exon combinations 7/4, 7/6, 7/8 and 7/9, the smallest product ever observed (Figure 2a). Since these results were obtained repeatedly using independent RNA preparations, we do not think that these accessory products resulted from cross-contamination or product carry-over but rather from alternative splicing of the exon 9/4 fusion product. Two patients (nos. 21 and 22; 7%) showed fusions of *EWS* exon 7 and *ERG* exon 6. Therefore, a total of 93% of all tumour-derived samples were positive by RT-PCR either for *EWS/FLI*-1 or *EWS/ERG* rearrangements.

b

| *EWS* ex 9 | *FLI*-1 ex 4 |
|---|---|
| TTCAATAAGCCTGGTG | ACCCCACACTGTGGACA |

| *EWS* ex 7 | *FLI*-1 ex 4 |
|---|---|
| AGCTACGGGCAGCAGA | ACCCCACACTGTGGACA |

| *EWS* ex 7 | *FLI*-1 ex 6 |
|---|---|
| AGCTACGGGCAGCAGA | ACCCTTCTTATGACTCA |

| *EWS* ex 7 | *FLI*-1 ex 8 |
|---|---|
| AGCTACGGGCAGCAGA | ATCCGTATCAGATCCTG |

| *EWS* ex 7 | *FLI*-1 ex 9 |
|---|---|
| AGCTACGGGCAGCAG | GAAGCGGGCAGATCCAG |

**Figure 2** **a**, Nested RT-PCR analysis of a cell line derived from a metastasis from patient no. 2 (right lane). Arrows point to amplification products that turned out to be derived from *EWS/FLI*-1 chimaeric RNAs after nucleotide sequencing. *EWS/FLI*-1 exon combinations are indicated. All other bands resulted from amplification of either non-specific sequences, as is often observed in nested PCR, or/and heteroduplex DNA formed between the specific alternative products. Left lane, size marker **b**, Results from sequence analysis of excised PCR products resulting from alternative splicing.

In contrast, routine cytogenetics revealed chromosome 22 rearrangements in only 18 out of 27 tumours (67%), partly because of failure to obtain mitotic figures (5/9) and partly because of insufficient tumour-derived material (4/9). Fifteen tumours revealed a t(11;22)(q24;q12) and one tumour a del22q. In all of these cases an *EWS/FLI*-1 fusion was demonstrated by RT-PCR. In one tumour (patient 10) displaying a t(2;22)(p25;q12), no fusion transcript could be detected although control amplifications of $MIC2^{p30-32}$ confirmed satisfactory quality of the RNA samples. This tumour was a typical $MIC2^{p30-32}$-positive chest wall tumour evolving from the right ninth rib (Askin tumour) with pleural effusion and metastases to the lungs and to the liver. In contrast, another patient (no. 21) with t(2;22)(q35;q12) expressed an *EWS/ERG* chimaeric RNA.

One patient (no. 5), who repeatedly failed to demonstrate the presence of chimaeric tumour transcripts in RT-PCR using either *FLI*-1 or *ERG*-specific primers, had a tumour from the right femur reaching to the gluteal region with bone marrow involvement as investigated by light microscopy. The tumour stained positive for the $MIC2^{p30-32}$ antigen but was negative for desmine and several neuroendocrine markers (i.e. neuron-specific enolase, S100, ganglioside GD2, chromogranin). The tumour cell morphology in light microscopy was compatible with a diagnosis of atypical Ewing's sarcoma. The tumour karyotype was 48,XY,del(1)(p34),+6,+12 with cytogenetically normal pairs of chromosome 22, 21 and 11. In addition, Northern blot analysis did not reveal aberrant *EWS* transcripts or expression of rearranged *FLI*-1 RNA.

Finally, RNA extracted from all tumours with a diagnosis other than ET (i.e. three rhabdomyosarcomas, one lymphoma, one neuroblastoma, one osteosarcoma and one malignant melanoma) did not reveal any *EWS* chimaeric transcript in RT-PCR.

A comparison of the distribution of primary tumour sites with the different exon combinations revealed the following (Table III). The relative frequency of the most common *EWS/FLI*-1 exon combination 7/6 - type I - (19 tumours) was higher in tumours of the extremities (six legs, three arms) than in central axis tumours (four chest wall, three pelvis) or in tumours in other locations (three cases). In contrast *EWS* exon 7/*FLI*-1 exon 5 transcripts - type II - (four tumours) were present in tumours of the chest wall and the pelvis (two cases each) only. Studies of the extent of disease showed that 78% (14/18) of patients with localised disease expressed type I RNA. In contrast, type II transcripts predominated in patients with disseminated disease at diagnosis (three cases) as compared with localised tumours (one case). This patient (no. 7) with a thoracopulmonary ET achieved a partial remission after two courses of chemotherapy, but shortly thereafter showed a tumour progression with lung and bone metastases. This tumour displayed a p53 mutation, which is very rare in patients with ET (Kovar *et al.*, 1993), and this may have been responsible for the aggressive tumour growth.

In this study, *EWS* chimaeric transcripts were also detected in two patients with extraosseous ET (nos. 22 and 23). One tumour, evolving from the chest wall with secondary infiltration of the scapula, showed a fusion of *EWS* and *ERG* (no. 22). In the other case (patient 23), ET developed as a secondary malignancy from the iliopsoas muscle after acute lymphoblastic leukaemia and expressed a chimaeric transcript with *EWS* exon 7 fused to *FLI*-1 exon 7.

**Table III** Different exon combinations, tumour localisation and extent of disease

| | Chest wall | Pelvis | Upper extremities | Lower extremities | Others | Local disease | Metastases |
|---|---|---|---|---|---|---|---|
| *EWS* ex 7/*FLI-1* ex 6 | 4 | 3 | 3 | 6 | 3 | 14 | 5 |
| *EWS* ex 7/*FLI-1* ex 5 | 2 | 2 | 0 | 0 | 0 | 1 | 3 |
| Others | 1 | 0 | 0 | 1 | 1 | 2 | 1 |
| *EWS/ERG* | 1 | 0 | 1 | 0 | 0 | 1 | 1 |

### *Fusion transcripts and clinical outcome*

Response data to chemotherapy were available for all patients positive for *EWS* rearrangements. The clinical outcome could not be evaluated in two patients because they were lost for follow-up. Ten patients showed no evidence of disease after a median follow-up of 21 months. Six of these ten patients had tumours expressing *EWS* exon 7/*FLI*-1 exon 6. Eight patients died as a result of disease progression. In this subset of patients, no preference for any exon combination has been observed. Two patients in complete remission died because of septic complications after allogeneic bone marrow transplantation. Furthermore, six patients are still on therapy and so far were not analysed for disease survival. In view of the relatively short follow-up in most patients, these results have to be considered preliminary.

### Discussion

Owing to the lack of differentiation, cytologically distinguishing ETs from other small round-cell tumours of childhood and adolescence is often difficult. Among immunological markers only MIC2$^{p30-32}$ is consistently highly expressed on ET cells, but it is not limited to this group of malignancies. Because of the high incidence of chromosome 22q12 aberrations, cytogenetic demonstration of these rearrangements should be pivotal to the unambiguous diagnosis of ET. However, routine cytogenetic analysis is often hampered by non-growth of tumour cells *in vitro*. Moreover, complex rearrangements with and without involvement of chromosome 11 have been identified at the chromosomal level in approximately 9% of cytogenetically analysable ET cases (Turc-Carel *et al*., 1988). This observation might be partially explained by the orientations of the genes involved in the translocations: *EWS* and *FLI*-1 are orientated from the centromere to the telomere, whereas *ERG* is orientated from the telomere to the centromere (Crete *et al*., 1993). Presumably, in-frame fusion of *EWS* and *ERG* can only be accomplished by either interstitial or complex translocations involving other chromosomes. This hypothesis might explain the lack of cytogenetic evidence for chromosome 21 rearrangements in ETs and the 17% translocation negatives observed in earlier studies (Turc-Carel *et al*., 1988). In our cohort patients 21 and 22 expressing *EWS/ERG* fit into this category. The molecular demonstration of chimaeric *EWS* RNA expression by RT-PCR as exemplified in this study appears to be more reliable than classical cytogenetics for ET diagnosis. Chimaeric *EWS* transcripts were identified in 93% of all cases investigated. By contrast, routine cytogenetics displayed a chromosome 22 rearrangement in only 67% of all tumours analysed. Only in 2 out of 30 tumours (7%) could no *EWS* chimaeric transcript be revealed by RT-PCR and Northern blotting (data not shown). In one of these cases an *EWS* rearrangement was demonstrated by molecular cytogenetics, suggesting the involvement of another so far unidentified ETS-related gene (data not shown). The other tumour (patient no. 5) was negative for any chromosome 22q12 rearrangement. Because of the clinical and histopathological appearance this tumour was classified as 'atypical Ewing's sarcoma'. However, this term is poorly defined. Tumours assigned to this group should therefore be reclassified on the basis of *EWS/FLI*-1 or *EWS/ERG* expression. Thus, it cannot be excluded that this patient suffered from some neoplasm other than ET. The data presented in this report confirm the high degree of variability of *EWS* chimaeric transcripts and resultant PCR products in ETs reported earlier.

In this study, the percentage of tumours expressing either type I or type II transcripts was 82% (23/28), which is similar to the proportion (38/47) recently observed by Zucman *et al*. (1993). The data collected in our study from a relatively small number of cases suggest that the *EWS/FLI*-1 exon combination 7/6 predominates in fusion transcripts of localised tumours of the extremities, whereas the combination *EWS* exon 7/*FLI*-1 exon 5 was more frequent in metastatic disease and tumours of the central axis. However, the significance of these findings has to be carefully evaluated in much larger cohorts of patients. Moreover, we could demonstrate the presence of a t(11;22)(q24;q12) and a t(21;22)(q24;q12) in two cases of malignancies of soft tissues at the molecular level, thus confirming the diagnosis of extraosseous ET. One of these tumours, which occurred as a second malignancy after treatment of acute lymphoblastic leukaemia, expressed an *EWS/FLI*-1 exon 7/7 fusion transcript which has not been reported before and therefore appears to be rare. In another unusual case with a single defined gene rearrangement at the DNA level (data not shown), a large *EWS/FLI*-1 transcript including *EWS* exons 1-9 as well as *FLI*-1 exons 4-9 was coexpressed with a number of deduced splicing variants, all lacking *EWS* exons 8 and 9 and fused to *FLI*-1 exons 4, 6, 8 or 9. In the protein encoded by the smallest product (*EWS/FLI*-1 exons 7/9) the *EWS* regulatory region is directly joined to the *FLI*-1 DNA-binding domain. This product has been predicted to occur as a parallel to the smallest *EWS/ERG* fusion observed by Zucman *et al*. (1993). The coexistence of alternative splicing variants seems to be common in cells carrying chromosomal translocations and has been demonstrated to occur in *BCR/ABL*-, *PML/RAR*- and *PBX1/E2A*-positive leukaemias (Shtivelman *et al*., 1986; Solomons *et al*., 1991; Izraeli *et al*., 1992). The possible presence of multiple differentially processed fusion transcripts in one tumour makes the application of additional methods for specification mandatory. We performed nested PCR as well as hybridisation to *FLI*-1- and *ERG*-specific probes and sequence analysis in order to distinguish specific amplification products from non-specific ones. Moreover, these very sensitive methods will be of particular help in the analysis of minimal metastatic and residual disease. The protocol applied in this report allowed detection of one tumour cell in $10^6$ nucleated cells by nested PCR in multiple mixing experiments (C. Pfleiderer *et al*., manuscript in preparation).

In conclusion, we think that comparison of molecular and clinical data as initiated in this report should be extended to much larger cohorts of ET patients since the results presented do not exclude a biological influence of the variable hinge region of *EWS* chimaeric oncoproteins on the clinical course of the disease in ET.

We would like to thank Gunhild Jug, Karin Kos, Bernadette Gruber and Silvia Bauer for excellent technical assistance. We also wish to thank the collaborating investigators of the Austrian Pediatric Oncology Group for the submission of clinical data. This study was supported in part by the 'Jubiläumsfonds der Österreichischen Nationalbank', Grant No. 4893, and by the 'Österreichische Kinderkrebshilfe'.

**References**

AMBROS, I.M., AMBROS, P.F., STREHL, S., KOVAR, H., GADNER, H. & SALZER-KUNTSCHIK, M. (1991). MIC2 is a specific marker for Ewing's sarcoma and peripheral primitive neuroectodermal tumours. *Cancer*, **67**, 1886–1893.

AURIAS, A., RIMBAUT, C., BUFFE, D., DUBOUSSET, J. & MAZABRAUD, A. (1983). Chromosomal translocation in Ewing's sarcoma. *N. Engl. J. Med.*, **309**, 496–497.

CHOMCZYNSKI, P. & SACCHI, N. (1987). Single-step method of RNA isolation by acid guanidinium thiocyanate–phenol–chloroform extraction. *Anal. Biochem.*, **162**, 156–159.

CRETE, N., DELABAR, J.M., RAHMANI, Z., YASPO, M.L., KRAUS, J., MARKS, A., SINET, P.M. & CREAU-GOLDBERG, N. (1993). Partial physical map of chromosome 21 from fibroblast and lymphocyte DNA. *Hum. Genet.*, **91**, 3, 245–253.

DELATTRE, O., ZUCMAN, J., PLOUGASTEL, B., DESMAZE, C., MELOT, T., PETER, M., KOVAR, H., JOUBERT, I., DEJONG, P., ROULEAU, G., AURIAS, A. & THOMAS, G. (1992). Gene fusion with an ETS-binding domain caused by chromosome translocation in human tumours. *Nature*, **359**, 162–165.

DOWNING, J.R., HEAD, D.R., PARHAM, D.M., DOUGLASS, E.C., HULSHOF, M.G., LINK, M.P., MOTRONI, T.A., GRIER, H.E., CURCIO-BRINT, A.M. & SHAPIRO, D.N. (1993). Detection of the (11;22)(q24;q12) translocation of Ewing's sarcoma and peripheral neuroectodermal tumour by reverse transcription polymerase chain reaction. *Am. J. Pathol.*, **143**, 1294–1300.

EMMINGER, W., EMMINGER-SCHMIDMEIER, W., HAWLICZEK, R., PETERS, C., HÖCKER, P. & GADNER, H. (1991). High-dose melphalan, etoposide +/– carboplatin (MEC) combined with 12-Gy fractionated total-body irradiation in children with generalized solid tumours. *Pediatr. Hematol. Oncol.*, **8**, 13–22.

IZRAELI, S., KOVAR, H., GADNER, H. & LION, T. (1992). Unexpected heterogeneity in E2A/PBX1 fusion messenger RNA detected by the polymerase chain reaction in pediatric patients with acute lymphoblastic leukemia. *Blood*, **80**, 1413–1417.

JÜRGENS, H., EXNER, U., GADNER, H., HARMS, D., MICHAELIS, J., SAVER, R., TREUNER, J., VONTE, T., WINKELMANN, W., WINKLER, K. & GÖBEL, V. (1988). Multidisciplinary treatment of primary Ewing sarcoma of bone. A 6-year experience of a European Cooperative Trial. *Cancer*, **61**, 23–32.

KOSCIELNIAK, E., TREUNER, J., JÜRGENS, H., WINKLER, K., BÜRGER, D., HERBST, M., RITTER, J., NIETHAMMER, D., MÜLLER-WEIHRICH, S., BERNHARD, G., KEIM, M. & KARDOS, G. (1991). Die Behandlungsergebnisse der Weichteilsarkome im Kindesalter-und Jugendalter: Ergebnisse der multizentrischen Therapiestudie. *Klin. Pädiatr.*, **203**, 1991.

KOVAR, H., DWORZAK, M., STREHL, S., SCHNELL, E., AMBROS, I., AMBROS, P.F. & GADNER, H. (1990). Overexpression of the pseudoautosomal gene MIC2 in Ewing's sarcoma and peripheral primitive neuroectodermal tumours. *Oncogene*, **5**, 1067–1070.

KOVAR, H., AUINGER, A., JUG, G., ARYEE, D., ZOUBEK, A., SALZER-KUNTSCHIK, M. & GADNER, H. (1993). Narrow spectrum of infrequent p53 mutations and absence of MDM2 amplification in Ewing tumours. *Oncogene*, **8**, 2683–2690.

MAY, W.A., GISHIZKY, M.L., LESSNIK, S.L., LUSFORD, L.B., LEWIS, B.C., DELATTRE, O., ZUCMAN, J., THOMAS, G. & DENNY, C.T. (1993). Ewing sarcoma 11;22 translocation produces a chimaeric transcription factor that requires the DNA-binding domain encoded by *FLI-1* for transformation. *Proc. Natl Acad. Sci. USA*, **90**, 5752–5756.

MANIATIS, T., FRITSCH, E.F. & SAMBROOK, J. (1982). *Molecular Cloning: A Laboratory Manual.* Cold Spring Harbor Laboratory Press: Cold Spring Harbor, NY.

OHNO, T., RAO, V.N. & REDDY, E.S. (1993). EWS/FLi-1 chimaeric protein is a transcritional activater. *Cancer Res.*, **53**, 5859–5863.

PLOUGASTEL, B., ZUCMAN, J., PETER, M., THOMAS, G. & DELATTRE, G. (1993). Genomic structure of the *EWS* gene and its relationship to EWSR1, a site of tumour-associated chromosome translocation. *Genomics*, **18**, 609–615.

SHTIVELMAN, E., LIFSHITZ, B., GALE, R.P., ROE, B.A., CANAANI, E. (1986). Alternative splicing of RNAs transcribed from the human abl gene and from the bcr-abl fusion gene. *Cell*, **47**, 277.

SOLOMONS, E., GODDARD, A. & BORROW, J. (1991). Molecular analysis of the t(15;17) in acute promyelocytic leukemia (abstract). *Haematologica*, **76** (Suppl. 4), 15.

SORENSEN, P.H.B., LESSNICK, S.L., LOPEZ-TERRADA, D., LIU, F.X., TRICHE, T.J. & DENNY, C.T. (1994). A second Ewing's sarcoma translocation, t(21;22), fuses the EWS gene to another ETS-family transcription factor, ERG. *Nature Genet.*, **6**, 146–151.

TURC-CAREL, C., AURIAS, A., MUGNERET, F., LIZARD SIDANER, I., VOLK, C., THIERY, J.P., OLSCHWANG, S., PHILIP, I., LENOIR, G.M. & MAZABRAUD, A. (1988). Chromosomes in Ewing's sarcoma. An evaluation of 85 cases and remarkable consistency of t(11;22)(q24;q12). *Cancer Genet. Cytogenet.*, **32**, 229–238.

WHANG-PENG, J., TRICHE, T.J., KNUTSEN, T., MISER, J., DOUGLASS, E.C. & ISRAEL, M.A. (1984). Chromosomal translocations in peripheral neuroepithelioma. *N Engl. J. Med.*, **311**, 584–585.

ZUCMAN, J., DELATTRE, O., DESMAZE, C., PLOUGASTEL, B., JOUBERT, I., MELOT, T., PETER, M., DEJONG, P., ROULEAU, G., AURIAS, A. & THOMAS, G. (1992). Cloning and characterization of the Ewing's sarcoma and peripheral neuroepithelioma t(11;22) translocation breakpoints. *Genes Chrom. Cancer*, **5**, 271–277.

ZUCMAN, J., MELOT, T., DESMAZE, C., GHYSDAEL, J., PLOUGASTEL, B., PETER, M., ZUCKER, J.M., TRICHE, T.J., SHEER, D., TURC-CAREL, C., AMBROS, P., COMBARET, V., LENOIR, G., AURIAS, A., THOMAS, G. & DELATTRE, O. (1993). Combinatorial generation of variable fusion proteins in the Ewing family of tumours. *EMBO J.*, **12**, 4481–4487.